# Barriers to community case management of malaria in Saraya, Senegal: training, and supply-chains

**DOI:** 10.1186/1475-2875-12-95

**Published:** 2013-03-14

**Authors:** Demetri A Blanas, Youssoupha Ndiaye, Kim Nichols, Andrew Jensen, Ammar Siddiqui, Nils Hennig

**Affiliations:** 1 Global Health Program, Icahn School of Medicine at Mount Sinai, One Gustave L. Levy Place, New York, NY 10029, USA; 2 District Health Center of Saraya, Senegalese Ministry of Health, B.P. 30, Kedougou, Region of Kedougou, Senegal; 3 African Services Comittee, 429 West 127th Street, New York, NY 10027, USA

**Keywords:** Artemisinin, Community health aides, Diagnostic test kits, Malaria

## Abstract

**Background:**

Health workers in sub-Saharan Africa can now diagnose and treat malaria in the field, using rapid diagnostic tests and artemisinin-based combination therapy in areas without microscopy and widespread resistance to previously effective drugs.

**Objective:**

This study evaluates communities’ perceptions of a new community case management of malaria programme in the district of Saraya, south-eastern Senegal, the effectiveness of lay health worker trainings, and the availability of rapid diagnostic tests and artemisinin-based combination therapy in the field.

**Methods:**

The study employed qualitative and quantitative methods including focus groups with villagers, and pre- and post-training questionnaires with lay health workers.

**Results:**

Communities approved of the community case management programme, but expressed concern about other general barriers to care, particularly transportation challenges. Most lay health workers acquired important skills, but a sizeable minority did not understand the rapid diagnostic test algorithm and were not able to correctly prescribe arteminisin-based combination therapy soon after the training. Further, few women lay health workers participated in the programme. Finally, the study identified stock-outs of rapid tests and anti-malaria medication products in over half of the programme sites two months after the start of the programme, thought due to a regional shortage.

**Conclusion:**

This study identified barriers to implementation of the community case management of malaria programme in Saraya that include lay health worker training, low numbers of women participants, and generalized stock-outs. These barriers warrant investigation into possible solutions of relevance to community case management generally.

## Background

Community case management of malaria (CCMm) consists of treating patients with prepackaged anti-malarial drugs that are distributed by members of their own community. This study examines a CCMm programme that relies on rapid diagnostic tests (RDTs) for diagnosis and artemisinin-based combination therapy (ACT) for treatment in the district of Saraya, south-eastern Senegal. It assesses the programme’s acceptance by communities in Saraya, the effectiveness of the lay health worker training, and the availability of supplies in the field.

The World Health Organization (WHO) estimates global malaria incidence in 2010 at 216 million cases, of which 81% were in Africa [1]. In the same year, mortality due to malaria is estimated to be from 655,000 to 1,238,00 deaths, 91% of which occurred in Africa and the majority among children under 5 years of age [1,2]. Many of these deaths also occurred in rural areas where there is sparse health infrastructure [3-5].

In 2005, Senegal’s national malaria control programme reported that malaria accounted for 28% of mortality in health facilities and was the single largest contributor to documented mortality [6]. *Plasmodium falciparum* accounts for the vast majority of infections, with fewer cases of *Plasmodium ovale* and *Plasmodium malariae*[7]. Deaths from malaria in Senegal are projected to have increased over three-fold between 1980 and 2000 (4,888 to 15,125 deaths) due to increasing population size, and to have decreased by over a third between 2000 and 2010 (15,125 to 10,150 deaths), likely due to intensified prevention and treatment efforts [2].

Community case management of malaria (previously known as home management of malaria) consists of a lay health worker with no formal health training providing malarial diagnostic or therapeutic care outside of a formal health care establishment [8]. The WHO recommends that CCMm be implemented in areas where a health facility is not accessible to the majority of people within 24 hours of illness onset [1]. As of 2010, 42 countries, including Senegal, have implemented some form of CCMm with RDTs [1]. Several studies have shown that CCMm is qualitatively acceptable to communities or providers [9-13]. Some researchers have assessed cure rates including a Ghana-, Nigeria-, and Uganda-based study that found greater than 90% parasitological cure rates in all sites where CCMm had been implemented [11]. Despite these positive findings, other authors have raised concerns about CCMm. Some have questioned the ability of lay health workers who need but cannot afford eyeglasses to read RDTs correctly [14] based on a high rate of untreated ophthalmologic conditions in sub-Saharan Africa [15,16]. Others have pointed out that the wide implementation of CCMm programmes is expensive and could accelerate the emergence of resistance to ACT [14]. There is also controversy around the impact of CCMm programmes on morbidity or mortality, with a Cochrane Review identifying only one randomized control CCMm trial showing a decrease in mortality (Ethiopia) [17,18]. Despite these concerns, CCMm with RDTs and ACT is endorsed by the WHO and widely accepted in areas where malaria is endemic and there is sparse health infrastructure [1].

Senegal’s national malaria control programme introduced ACT (artesunate-amodiaquine) as first-line treatment for uncomplicated malaria in 2006. In 2007, it began offering RDTs in most health centres, health posts, and operational health huts nationally [19]. A recent study found that the prescription of ACT decreased from 73% to 32% of all malaria-like febrile illnesses three years after RDTs had been introduced [19].

## Methods

### Study area

The study site is located in Saraya, a district in south-eastern Senegal that borders Mali and Guinea. The population is estimated at over 34,000, with a density of seven persons per square kilometre. Malaria is seasonally holoendemic in Saraya with highest incidence in the rainy season (June to November) [20].

### Community participants in the study

The study included two groups of lay health workers: a group of community health workers (CHWs) or *agents de santé communautaires*, and a group of community medicine distributors (CMDs), or *distributeurs de soins à domicile*. Each CHW works in a village with a functional health hut, which is a basic local health care facility in a village without a clinic. The CMDs work in villages without health huts. Of note, unlike the CHWs, the CMDs are trained only to manage uncomplicated malaria and are not permitted by the ministry of health to manage any other condition.

### Drugs and test used

The CCMm programme used loose-dose artesunate-amodiaquine ACT and the Paracheck Pf® RDT. This study assessed the functioning of the CCMm programme in Saraya through the following qualitative and quantitative methods: 1) Focus groups with villagers in CCMm villages, 2) pre- and post-training questionnaires with CMDs, and 3) post-training questionnaires with CHWs two months after their training. The study was originally designed to assess both the CHWs and CMDs with pre- and post-training questionnaires; however, weather, road conditions, and feasibility constraints prevented the research team from conducting pre- and post-training questionnaires for the CHWs limiting their evaluation to a post-training assessment. Although a deviation from the original research design, the different CHW and CMD assessment methods made possible an assessment of different aspects of the CCMm programme, specifically short-term knowledge gain from the training (the CMD pre- and post-training questionnaires), and long-term retention of that knowledge (CHW post-training assessment).

### Focus groups

Six focus groups were carried out in villages where the CCMm programme had already begun and selected participants through convenience sampling after meeting with the village chief and included any adult villagers willing and available to participate at the time of the visit to their village. The focus groups explored two predetermined domains: community priorities with respect to malaria, and local perceptions of the CCMm programme.

### Selection of lay health workers

The CHWs and CMDs were selected to participate in the CCMm programme independent of and prior to the initiation of the research study. Communities chose the CHWs to run the health hut in their villages, whereas village leaders chose the CMDs for the express purpose of the CCMm programme.

### Lay health worker trainings

Representatives of the national malaria programme designed and led the trainings in French and a local CHW translated into Malinké. The trainings lasted three days and included five modules, each of which included lectures, question-and-answer sessions, role-playing, and job-aids. Subjects included the aetiology of malaria, signs and symptoms, high-risk group identification, behaviour change, and the programme protocol. The CHWs and CMDs were trained in May and August 2009 respectively.

### CMD assessment

The national malaria control programme wrote the 10-item CMD pre- and post-training questionnaire. The research team administered the questionnaire to all 26 CMDs who participated in the training. The questionnaire assessed three domains: 1) Their understanding of the transmission and prevention of malaria, 2) its clinical presentation, and 3) their understanding of the CCMm protocol. Because six CMDs could not read or write, a designated CHW translator helped them complete the questionnaire.

### CHW assessment

The research team designed two questionnaires to assess the training of the CHWs. One questionnaire assessed the functioning of the CCMm programme at the CHW health huts, and the other assessed CHW knowledge of information taught in the CCMm programme training. The surveys were based on themes that emerged in the focus groups and piloted them with district health staff. The difference in the evaluation tools for CMDs and CHWs was a consequence of unforeseen poor weather and road conditions, which forced the research team to deviate from their original plan of carrying out pre- and post-training questionnaires for both groups of lay health workers.

### Data management

The focus group moderator collected qualitative data by taking notes of themes and quotes that emerged during focus groups. The quantitative data was collected on written surveys and entered into a Microsoft® Excel file and stored on a hard drive with password protection.

### Data analysis

The research team analyzed the qualitative data (focus groups) using the constant comparisons analytic process and in-vivo codes based on grounded theory methodology [21]. Basic data analysis was carried out with MS Excel®, and statistical analysis with SPSS®. The analysis of the 10-item questionnaire administered to the CMDs to compare pre- and post-training scores was done with paired T-tests. The CHW and CMD groups were not compared due to different baseline levels of training and to the different assessment methods.

### Ethical considerations

The Institutional Review Board of the Icahn School of Medicine at Mount Sinai approved this research (reference number: 09-0925) as being in accordance with the Helsinki Declaration. Local community approval was also obtained for this study. Oral informed consent was obtained for all lay health worker questionnaires and surveys.

## Results

### Qualitative findings

Six focus groups with 42 total participants with approximately equal numbers of men and women participants were performed (Table 1). Four major themes emerged in the community priorities domain: medication and RDT shortages or stock-outs, transportation difficulties with inability to complete referrals made by CHWs, community control, and problems accessing health posts. All groups cited barriers to transportation and referral completion due to poor roads and distance as the primary barrier to the CCMm programme. The quotation by a 45-year-old gentleman illustrates this common complaint.

“*The nurse at the health post blames us peasants for bringing in patients late, but how can we carry a sick person on our head for 15 kilometres? […] The whole problem is that if a child is sick […] or if a woman is pregnant and sick - you can’t carry her on your back either. How can you bring them to the health post? […] This is very, very hard - especially if the river and swamp is overflowing. Then you simply have to put your life in god’s hands and stay at home*.”

**Table 1:** Participant demographics

		**Total n**	**Gender n (%)**		**Mean age**
			**Female**	**Male**	
Focus groups	Villagers	42	18 (43%)	24 (57%)	43
Surveys	CHWs	26	5 (19%)	21 (81%)	31
	CMDs	26	2 (8%)	24 (92%)	28

**Figure 1:**
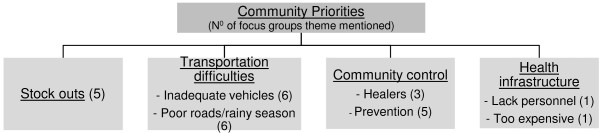
Focus groups major themes.

Five focus groups cited ACT stock-outs as a major weakness. Most participants emphasized community control in fighting malaria, including consulting traditional healers (three groups), and community prevention with insecticide-treated nets and the burning of local plants to deter mosquitoes (five groups). One focus group also complained of insufficient health personnel and facilities and a high cost of care (Figure 1). With regards to community perceptions of the CCMm programme, participants expressed both skepticism and confidence in the CCMm programme as illustrated by the following quotation by a 60 year-old village woman,

“We are doubtful that it will work because we have been told that many programs like this will happen in the past, but they often never end up happening.”

Whereas an elderly gentlemen felt that,

“Progress can be made in the community’s fight against malaria. Look at Guinea Worm and how the community got rid of that.”

### Quantitative findings

Both CHWs and CMDs were predominantly male and young adults (Table 1).

#### CMD pre- and post-training questionnaires

Twenty-six CMDs participated in the training and all completed a written pre- and a post-training 10-question evaluation (Table 2). The mean overall score on this evaluation rose from 52% before the training to 73% after (p = 0.02), and the proportion of CMDs who attained a score indicating that they understood and could carry out the protocol rose from 52% to 75% (p < 0.01). An increase in the proportion of CMDs able to recognize the clinical presentation of malaria was not statistically significant (37% vs. 47%, p = 0.21).

**Table 2:** Community medicine distributer pre- and post-training questionnaire scores

	**Pre-training score**	**Post-training score**	**P-value**
Overall understanding and ability to carry out protocol	14 (52%)	20 (75%)	P < 0.01
Able to recognize clinical malaria symptoms	10 (37%)	12 (47%)	P = 0.21
Overall questionnaire	14 (52%)	19 (73%)	P = 0.02

#### CHW health hut questionnaire

The main findings were that RDTs and ACT had expired or run out in most villages, that CHWs generally kept accurate patient logs and that all CHWs performed malaria tests free of charge and sold treatment at the price set by the health ministry, as stipulated by the CCMm protocol (Table 2).

#### CHW knowledge questionnaires

While most CHWs answered most questions correctly, only half could correctly explain the programme’s referral algorithm even when showed the visual depiction of the algorithm. For no topic did more than about three quarters of CHWs answer all questions correctly. Areas where the greatest number of CHWs answered correctly included transmission of malaria by mosquitoes, correct identification of signs and symptoms of complicated and uncomplicated malaria, correct identification of infants and pregnant women as most vulnerable groups, and of insecticide-treated nets as a prevention measure (Table 3).

**Table 3:** Community health worker assessment

***Field visits. n = 19***		**n (%)**
Villages where RDTs were expired or unavailable on day of visit		14 (74%)
Villages where ACT was expired or unavailable on day of visit		13 (68%)
CHW workers who maintained an accurate register		16 (84%)
CHW that maintained a supply stock management sheet		9 (48%)
Villages where RDTs were provided free of charge		19 (100%)
Villages where pricing of ACT was correct		19 (100%)
Villages where CHWs organized educational activities on malaria		11 (58%)
*Knowledge assessment and questionnaire. n = 26*		
Knows that malaria is transmitted by mosquitoes		20 (76%)
Correctly identifies signs and symptoms of uncomplicated malaria		18 (68%)
Correctly identifies signs and symptoms of complicated malaria		19 (72%)
Knows correct ACT dosing		16 (60%)
Correctly interprets RDT and referral algorithm		13 (50%)
Correctly identifies most vulnerable groups	Foreign	1 (4%)
	Pregnant women	18 (68%)
	Less than 2 months of age	20 (76%)
Correctly identifies prevention measures	Early treatment	3 (12%)
	Intermittent Preventive Treatment - SP	11 (44%)
	Sanitary environment	11 (44%)
	Insecticide-treated nets	19 (72%)

Legend: *SP*: sulphadoxine-pyrimethamine.

## Discussion

This qualitative and quantitative study has three principal findings with regards to acceptation of the CCMm programme by local communities, training of lay health workers, and the functioning of the CMMm programme in the field.

### Community perceptions of CMD programmes

First, communities in CCMm villages generally felt that the programme meets a real need by increasing access to RDTs and ACT, but expressed concerns that it will not solve the challenges of transporting patients that often make it difficult or impossible to complete referrals made by lay health workers. It is important to note that the challenge of transportation is an inherent limitation of any CCMm programme in a remote setting and cannot be addressed without major infrastructure improvements. This is in contrast to other aspects of malaria control such as lay health worker training and supply chain management, that are inherent components of CCMm and that can be improved to optimize the functioning of the programme as discussed below.

A second major qualitative theme that emerged from the focus groups was the importance of community control in efforts against malaria through traditional modalities and prevention efforts including insecticide-treated nets. Other analyses of community acceptance of CCMm programmes have also found appreciation by communities due to increased proximity of care and decreased cost [9-11]. One multi-site feasibility study found that communities criticized CCMm programmes for lack of lay health worker training, and that lay health workers are not compensated for their work [11], although these concerns were not voiced in this study’s focus groups.

### Lay health worker training

This study’s second major finding concerns the effectiveness of the lay health worker training, which was evaluated in two ways: through pre- and post-training questionnaires of CMDs, and on-site post-training questionnaires of CHWs. Although the differing evaluation mechanisms preclude any comparison of the CMDs and CHWs, they do allow for a short- and long-term evaluation of the lay health workers knowledge. In the short term, pre- and post-training questionnaires among CMDs demonstrated significant improvement in scores. This is consistent with a study in Laos, which found a less than 2% RDT interpretation error rate by lay health workers one year after a one-hour training [22]. A Zambia-based study similarly found lay health workers performed RDTs with 90% accuracy and with 93% correct result interpretation after a three-hour training assisted by visual job aids and RDT package inserts [14].

Although the CMDs’ scores improved statistically after training, this score cannot be correlated with field performance. This is highlighted by results of the CHW questionnaire performed in the field two months after their training, which showed that half of the CHWs could not interpret the RDT algorithm correctly and almost half could not prescribe ACT correctly. In fact, none of the women CHWs who had been trained in the CCMm programme were able to correctly interpret the RDT algorithm or prescribe ACT in the field on the post-training questionnaire. This is likely due to the fact that few women CHW participated in the CCMm programme in their villages after they were trained. The low number of women participating in the CCMm programme could have implications for the success of the programme as women (mothers) were the providers of care in the only CCMm randomized trial to have shown a statistically significant mortality decrease [17,18].

The CHWs performed well in other areas including knowledge of the aetiology of malaria, identifying its signs and symptoms, identifying infants and pregnant women as highest risk groups, and insecticide-treated nets as a prevention measure. Poor performance in the CHW post-training questionnaire included identification of foreigners as high-risk, and of removing standing water and debris, intermittent preventative treatment, and early treatment as important prevention measures.

### Availability of supplies

Finally, the assessment of CCMm functioning in the field revealed that ACT and RDTs had not been available since soon after the CHWs had completed their training two months prior to the assessment. The cause of the stock-out was not determined, however the programme manager in Saraya indicated that supplies were not available from the regional pharmacy and that the district health centre was not distributing ACT or RDTs to lay health workers as they then would not have enough supplies for the district health center. Recent assessments of ACT availability in other African countries have found similar evidence of wide-spread stock-outs [23-25]. A study of anti-malarial availability in six-African countries found rates of ACT stock-outs among CHWs ranging between 20% to 70%, although the authors do not provide a reason for the shortages [23]. A Malawi-based study identified a three-month per year stock-out of anti-malarial medications in eight centrally located government health centres that it attributed to inaccurate record-keeping and ignored supply requests [25]. Two Kenya-based studies documented ACT stock-outs due to procurement system failures and inadequate central stores, although the authors do not specify what caused these problems [24,26]. The Global Fund has implemented an initiative to increase the availability of ACT, the Affordable Medicines Facility, but that does not include Senegal in its first phase of implementation [27]. This initiative focuses on distribution within countries as well as production and may provide insight on how to increase the availability of ACT in other countries including Senegal. Although the findings of this study are limited to Saraya, they highlight the need for further investigation into the frequency, prevalence, and causes of stock-outs in Senegal.

### Limitations

The chief limitation of this study is that no medical outcome was measured. While the chief goal of any anti-malaria programme must be to reduce malaria, the incidence, prevalence and mortality of malaria in Saraya were unknown both before and after the implementation of the CCMm programme. Monitoring such outcomes would require the design and creation of an epidemiologic monitoring and reporting system in Saraya. Other variables which could be of interest to observe in an evaluation of a CCMm programme that were not measured in this study include time from malarial symptom onset to first dose of treatment, morbidity and mortality changes due to reduction in the misdiagnosis of other febrile childhood illnesses as malaria, and days of work lost due to clinic visits.

Another major limitation is the different evaluation tools used to assess the CMDs and CHWs. The original study design was to evaluate both groups of lay health workers with pre- and post-training questionnaires; however, weather and resulting poor road conditions prevented the research team from being present at the CHW training. Further feasibility constraints precluded visiting the CMDs two months after their training to assess long-term knowledge retention, as was done with the CHWs. Although a major limitation of this study, the two different evaluation tools have the advantage of representing both short- and longer-term assessment of the training’s effectiveness.

Finally, regarding the evaluations of CHW knowledge and performance, two caveats are in order. First, the CHWs knew they were being evaluated during the surveys, which may have influenced their behaviour and responses. Second, the CMDs were evaluated with written pre- and post-test questionnaires even though several of them were illiterate and required assistance from CHWs.

## Conclusion

Modeling suggests that there has been a significant decrease in malaria mortality in Senegal over the past ten years possibly associated with intensive malaria control efforts including increased access to ACT and RDTs through CCMm programmes [2]. This study assessed the acceptability and functioning of a CCMm programme in the field in a single district in Saraya, Senegal. It found generally good acceptance by communities due to perceived increased access to malarial treatment. It also found that although most lay health workers acquired necessary knowledge and skills through the training, a sizeable minority was unable to correctly interpret the RDT algorithm or prescribe ACT several months after their training. It also found that women CHWs few women were trained in the CCMm programme and those who were trained subsequently did not participate in their villages. Finally, this study found that three quarters of CCMm sites had stock-outs of RDTs and two thirds had stock-outs of ACT two months after the initiation of the programme due to a regional shortage. This study’s findings are limited to the district of Saraya, but highlight important considerations for CCMm programmes in general including CHW training, and supply chain management.

## Abbreviation

ACT: Artemisinin-based combination therapy; CCMm: Community case management of malaria; CHWs: Community health workers; CMDs: Community medicine distributors; RDTs: Rapid diagnostic tests; WHO: World Health Organization.

## Competing interests

The authors have no competing interests to declare.

## Authors’ contributions

DB, YN, and NH conceived and designed the study conception and carried out the data analysis and interpretation. DB and YN designed the research tools and conducted the site visits, focus groups, and administered the surveys. AJ, AS, MM, and KN made substantive contributions to data interpretation, and were integrally involved in revising the manuscript. NH also gave final approval for publication of the manuscript. All authors read and approved the final manuscript.
